# A Case of Gun Shot Wound Involving the Colon, Small Intestines and Right Kidney, Attended by Recovery

**Published:** 1841-07

**Authors:** John W. Richardson

**Affiliations:** Rutherford County, Tennessee


Art. III.—*A Case of Gun Shot Wound involving the Colon, Small Intestines and Right Kidney, attended by recovery:* Reported to the Medical Society of Tennessee, at its annual meeting in May, 1841: By John W. Richardson, M. D., of Rutherford County, Tennessee.

On the 26th of last November, I was called, in company with Dr. Watson, to visit Mr. K., a young man who had shot himself about 9 o'clock in the morning. On examination two hours after the wound was received, we ascertained that the ball, which weighed ʒiiss, had entered the abdomen on the right of the median line, and passed out about midway between the last rib and the sacro-iliac junction, immediately on the right of the spine.

Patient composed and very pale—abdomen natural—extremities cold—pulse regular and small—no hemorrhage. A plaster of simple cerate was applied to the wounds, and a poultice of wheat bran and ground mustard applied to the abdomen and right side, reaching even to the spine. A warm blanket was placed over him, and we awaited the consequences of the injury. At 2 P. M. he discharged nearly a pint of urine, the latter half of which was almost black with blood. At 4 o'clock, he passed urine again, but no more blood.

27th. Saw the patient at 9 A. M. Pulse 110, soft and small, though very regular while he is resting *perfectly quiet.* Abdomen becoming tense and swollen. Slept some during the latter part of the night. Complains much now of pain in the abdomen and back. Urine discharged regularly since yesterday evening, and of a healthy character. Gave two enemas, which were passed off very slowly without any fæces. Treatment the same, only discontinue the mustard. The poultices to be applied every six hours.

28th. Saw the patient at noon. Abdomen more tense and swollen, attendedwith considerable soreness and pain, which were greatly increased by the slightest pressure. Pulse 100, small though pretty firm. On moving him from his back to his side, his pulse sunk and became weak and irregular. Slept some in the latter part of the night—great thirst—turning from his side to his back, or vice versa, produces great sickness and disposition to syncope;—rests easiest on his back, and easier on the right side than on the left. Gave an enema made of warm water and strong soap, which was discharged very slowly, with much flatus, but feculent matter. The discharge of so much gas afforded some relief, and I *thought* somewhat reduced the swelling and tension of the abdomen, but whether it really did so I am not certain. Urine discharged regularly. Ordered a tea spoon full of spts. nit. dulc. every two hours, and 12 drops tinct. digitalis every four hours; same dressing continued.

29th. Slept some since yesterday, though suffered very much with pain in the abdomen and back all night; pulse 100, regular, and perhaps not quite as firm as on yesterday; abdomen, if any difference, more tense and swollen; tongue coated with a white fur in the middle, the fore part and edges being clean and not redder than usual; thirst still very great. Same treatment continued.

30th Noon. Slept more; tension and swelling somewhat subsided: pulse 90; tongue as yesterday; eyes and skin quite yellow; great thirst; wounds beginning to slough. Continue the treatment.

Dec. 1st. Swelling, tension and soreness all subsiding; slept some; pulse 90, soft and regular; not so much thirst; skin and eyes still yellow; urine healthy and discharged at the usual intervals. The spts. nit. and digitalis partially discontinued. Same dressing continued.

5th. Patient has remained in pretty much the same condition since the first. Fur on the tongue whiter and longer; yellowness of skin and eyes has disappeared; urine still healthy. Same dressing continued.

6th and 7th. General condition improved. Poultice discontinued, as its weight produced some uneasiness, and the abdominal swelling had nearly subsided. Spts. nit. and digitalis also discontinued. Urine healthy and discharged regularly. As he still seemed quite thirsty, I ordered a tea spoon full of cream tartar to a glass of sweetened water, to be taken as often as desired; a plaster of simple cerate to be kept over the wounds.

8th and 11th. Patient still improving; posterior wound nearly healed; anterior healing kindly.

12th. Enema given, which brought off a large, healthy feculent discharge, without any pain.

13th and 16th. Patient still improving; On the 16th my visits were discontinued, and on the 5th of January he rode home, a distance of four miles, and had, to all appearance, entirely recovered.

*Remarks.*—The patient ate his breakfast as usual; immediately afterwards had a large evacuation from his bowels, and returned to the house and shot himself. We were not able to trace the wound even into the abdominal cavity, a circumstance which I ascertained upon examination, after the failure, to have been produced altogether by a change of position. For I went to the spot where he was found upon the report of the pistol, and soon discovered that he was *sitting* when he shot, although he was found by the first attendant standing on his feet.

The ball entered the abdominal cavity one inch above a line drawn from the umbilicus to the anterior superior spinous process of the right ilium, and came out at the point before mentioned, viz: about midway between the last rib and the sacro-iliac junction; and I infer that it cut the colon the small intestines, and passed through the right kidney. This was my opinion at the time I first examined the case, and I have found no cause to change it. If the small intestines were very full, and the colon entirely empty, in some conditions of the body the colon might have escaped, though in the present case I think it did not. About two hours and a half after the injury, the patient threw up his breakfast but nothing else that I could discover. There was never any escape of gas, or feculent matter from the wound.

That the ball cut the kidney, I regard as positively certain. Its exit allowing it to have pursued a straight course in its passage, puts this point beyond dispute. And even if a doubt still existed, it seems that the *blood* discharged with the urine, in addition to the course of the ball, would dispel the doubt. The question might be asked, why was there no more blood discharged after the first urination? And *another* might be asked by way of answer, why, if the kidney was not wounded, was any discharged at all? and from whence did it come? But there can be no doubt that the blood did come from the kidney, the upper half of which was lacerated; and it is probable that a coagulum formed in the pelvis and prevented the entrance of any more into the ureter.

The treatment was of the simplest character; for the case was one of that class in which little is left for the surgeon or physician to accomplish. The patient was a young man in good health, and perhaps in as fine a condition to recover from such an injury as we can imagine. The dressing was simple, and yet seemed to be all that the case required. The pulse was of the kind we usually find in gun shot wounds, where the intestines are injured, and such as attends the wounding of the abdominal viscera generally. The extremities were cold; another circumstance always present in such injuries.

While our patient lay still and quiet, his pulse was quite firm and 100 in a minute, yet upon the least exertion it became feeble, soft and irregular. In all such cases, when any doubt exists as to the propriety of blood-letting, it is advisable that the patient be gently moved; even make him change his position in bed, and if the pulse assumes the character above noticed, bleeding would be injurious. At the time when the inflammation was progressing so rapidly I should perhaps have used local bleeding, but it was not practicable to procure leeches, and there was so much soreness over the whole abdomen that cupping would have increased, rather than mitigated the evils anticipated.

The condition of the bowels presents us with an important principle in the treatment of such cases. Two enemas were given during the day after the injury, and one on the following day; yet no feculent discharge was produced. I then became satisfied that the lower bowels were emptied by the evacuation just before the injury was committed, and did not use the syringe any more until the 12th of December, seventeen days after the injury, when a copious and healthy stoool was produced. This course was adopted for the purpose of permitting the intestines to remain perfectly quiet, in order that nature might, if possible, repair the injury. The patient was not allowed any article of diet during his confinement, except milk, and apple water, or some such drink.

The yellowness of the skin and eyes on the 5th day produced some uneasiness, but in a short time it disappeared, and no appearance of biliary derangement was manifested, unless the condition of the tongue indicated it.

I cannot but believe that if V. S. to any extent had been practised, or the daily use of the syringe resorted to, a greater prostration of the vital organs would have been produced, and an irritable state of the bowels brought on, which would have placed the patient in a much worse, if not in a hopeless condition. The case, however, has taught me a valuable lesson; and that is, that in cases, when so little remains to be done, not to bring business on my hands by *doing too much;* nor to give remedies in anticipation of what *may* happen. In this case, we witness also the great restorative powers of Nature. Not an unfavorable circumstance, considering the extent of the injury, occurred from the first, and such as did appear were much milder than were to have been expected; and some of the most alarming symptoms usually attendant upon such extensive injuries did not occur at all.

